# Proteome identification of the silkworm middle silk gland

**DOI:** 10.1016/j.dib.2016.01.053

**Published:** 2016-02-04

**Authors:** Jian-ying Li, Lu-peng Ye, Jia-qian Che, Jia Song, Zheng-ying You, Shao-hua Wang, Bo-xiong Zhong

**Affiliations:** a Institute of Developmental and Regenerative Biology, Hangzhou Normal University, Hangzhou 310036, PR China; b College of Animal Sciences, Zhejiang University, Hangzhou 310029, PR China

**Keywords:** *Bombyx mori*, Middle silk gland, Silk protein synthesis, Shotgun proteomics, Label-free

## Abstract

To investigate the functional differentiation among the anterior (A), middle (M), and posterior (P) regions of silkworm middle silk gland (MSG), their proteomes were characterized by shotgun LC–MS/MS analysis with a LTQ-Orbitrap mass spectrometer. To get better proteome identification and quantification, triplicate replicates of mass spectrometry analysis were performed for each sample. The mass spectrometry proteomics data have been deposited to the ProteomeXchange Consortium (Vizcaíno et al., 2014) [1] via the PRIDE partner repository (Vizcaino, 2013) [2] with the dataset identifier PXD003371. The peptide identifications that were further processed by PeptideProphet program in Trans-Proteomic Pipeline (TPP) after database search with Mascot software were also available in .XML format files. Data presented here are related to a research article published in Journal of Proteomics by Li et al. (2015) [3].

**Specifications Table**

**Table:** 

Subject area	*Biology*
More specific subject area	*Insect proteomics*
Type of data	*Excel data sheets with identified proteins and corresponding peptides from each analyzed sample.*
How data was acquired	Ettan MDLC nanoflow/capillary LC system (GE Healthcare, Pittsburgh, PA) coupled with a LTQ mass spectrometer (Thermo Fisher Scientific) with a nano-electrospray ionization (ESI) source.
Data format	Analyzed
Experimental factors	No sample pretreatment applied.
Experimental features	The sample proteomes were fractionated using 1D SDS-PAGE followed by tryptic digest. Digested peptides were fractionated using MDLC system prior to LC–MS/MS. Data mining of the acquired MS output was performed by bioinformatics analysis.
Data source location	*Hangzhou, China*
Data accessibility	Data are available with this article and related to [3].

**Value of the data**

• High-confidence proteome identifications of the silkworm middle silk gland.
• The identified tissue-specific proteins are valuable for understanding of the functional differentiation among different regions of middle silk gland.
• Label-free quantitation of the three regions of silkworm middle silk gland to determine their relative abundances.
• In-depth proteome comparison with posterior silk gland will contribute to better understanding of the mechanism of silk protein synthesis.

## 1 Data, experimental design, materials and methods

In order to disclose the mechanism of high efficient synthesis of silk proteins, in-depth proteomic analysis of the silkworm middle silk gland (MSG) was performed with shotgun LC–MS/MS. The silkworm MSGs at the 3rd day of the fifth instar were dissected and cut into anterior (MSG-A), middle (MSG-M), and posterior (MSG-P) sections. The proteins were separated by 1D SDS-PAGE followed by in-gel trypsin digestion. The digested peptides were analyzed using a Nano-LC–MS/MS system with a LTQ-Orbitrap mass spectrometer. The generated raw MS data were deposited to the ProteomeXchange Consortium [1] via the PRIDE partner repository [2] with the dataset identifier PXD003371. We finally identified 643, 594, and 823 proteins from the MSG-A, -M, and -P, respectively, with a FDR of lower than 0.5% [3]. The differential expression of proteins was analyzed with a label-free quantification method. The differentially expressed proteins were further subjected to functional enrichment analysis (Fig. 1).

### 1.1 Sample preparation

The silkworm MSGs at the 3rd day of the fifth instar were dissected in pre-cooled physiological saline under a dissecting microscope. Each MSG was cut into three sections at the two turnings. To remove the secreted sericin proteins in the gland lumen, the MSG sections were immersed in pre-cooled 60% ethanol for 1–2 min to stiffen the sericins and draw them out with nippers. The protein extraction protocol was according to the description in our previous articles [4], [5]. Briefly, the tissues were mechanically homogenized on ice in lysis buffer that containing 2.5% SDS, 10% glycerin, 5% β-mercaptoethanol, and 62.5 mM Tris–HCl pH 6.8. The homogenate was further subjected to sonication treatment in an ice-bath. The protein concentration was determined by using the 2-D Quant Kit (Amersham Biosciences, Piscataway, NJ, USA) according to the manufacturer׳s instructions.

### 1.2 Protein separation and in-gel digestion

Totally 200 μg of proteins for each sample were loaded into four gel lanes and separated by SDS-PAGE using a 12.5% resolving gel with constant current of electrophoresis at 10 mA for 0.5 h and 12 mA for 1.5 h. After Coomassie Brilliant Blue (CBB) staining, the gel lanes were sliced into pieces and well washed with Milli-Q water. In-gel digestion of the protein samples was performed according to previous reports [4], [6]. The generated peptides were dried using the Savant SPD131DDA SpeedVac Concentrator (Thermo Fisher Scientific, Waltham, MA, USA) and stored at −20 ^o^C for the following use.

### 1.3 LC–MS/MS analysis

The dried peptides were re-suspended with 0.1% methanoic acid (Sigma). Five microliters of the digested peptides for each run were subjected to a nano-LC–MS/MS system with a linear ion trap Orbitrap mass spectrometer (LTQ-Orbitrap, Thermo Fisher Scientific, Bremen, Germany). The nanoflow/capillary LC system (GE Healthcare, Pittsburgh, PA, USA) equipped with a trapping column [PepMap C18, 300 μm i.d. ×5 mm, 3 μm, 100 Å (P/N160454), Sunnyvale, CA, USA], and a nanocolumn [Pep-Map C18, 75 μm i.d. ×15 cm, 3 μm, 100 Å (P/N160321), Sunnyvale, CA]. The peptides were firstly preconcentrated and washed on the C18 trap column, and then were separated on the analytical column with a 70 min gradient of buffer B (84% acetonitrile, 0.1% methanoicacid in water) from 5 to 45% and a 20 min gradient from 45 to 95% at a 300 nL/min flow rate. The LTQ-Orbitrap equipped with a nanospray source was used for the MS/MS experiment in the positive ion mode and was operated in a data-dependent mode using Xcalibur software. The MS scan range was 300–2000*m*/*z* with a resolution *R*=60,000 at *m*/*z* 400. The temperature of the ion transfer capillary was set at 160 °C and the spray voltage was 3.0 kV. Collision-induced dissociation (CID)‍ was conducted with an isolation width of 2 Da, normalized‍ collision energy of 35%, and activation q of 0.25 for MS/MS ‍acquisition. The five most intense ions were isolated for CID‍ fragmentation and measured in the linear ion trap with the‍ dynamic exclusion settings: repeat count 2, repeat duration 30 s,‍ exclusion duration 180 s. Triplicate replicates were performed for ‍each sample.‍

### 1.4 Protein identification, validation and quantification

MS/MS spectra were extracted from the raw data files by Mascot Deamon (Matrix Science, London, U.K.; 2.2) with the extract_msn program (version 2.07). The resultant .mgf files were searched against an in-house database on a local Mascot server (Matrix Science; 2.2). The database was consist of the 14623 predicted protein sequences coded by silkworm genome and 1739 annotated sequences from NCBI. The MS and MS/MS mass tolerances were 50 ppm and 0.6 Da, respectively, and up to two missed cleavages were permitted for fully tryptic peptides. Carboxamidomethyl modification on cysteine and oxidized modification on methionine were set as fixed and variable modifications, respectively. The resultant .dat files were subjected to Trans-Proteomic Pipeline (v4.0 JETSTREAM rev 2) for further validation process using PeptideProphet and ProteinProphet programs with the probability thresholds at 0.7 and 0.9, respectively. The false discovery rate (FDR) of identifications was evaluated by using a target-decoy search strategy [7]. The above parameter settings could control the FDR of protein identifications lower than 0.5%. The proteins identified with at least two assigned peptides were acceptable. For the proteins assigned in one group with common peptides, only the annotated proteins were kept in final identification list (Supplementary Table 1).

To screen the differentially expressed proteins, a label-free quantification method was used with the APEX program [8]. The ProteinProphet result XML file was submitted as one of the input files for computing the protein APEX score. The FPR cutoff of protein list was set at 0.01, and differential protein expression between two samples was considered as significant when the *p*-value is less than 0.05 with at least 2-fold changes.

## Figures and Tables

**Fig. S1:**
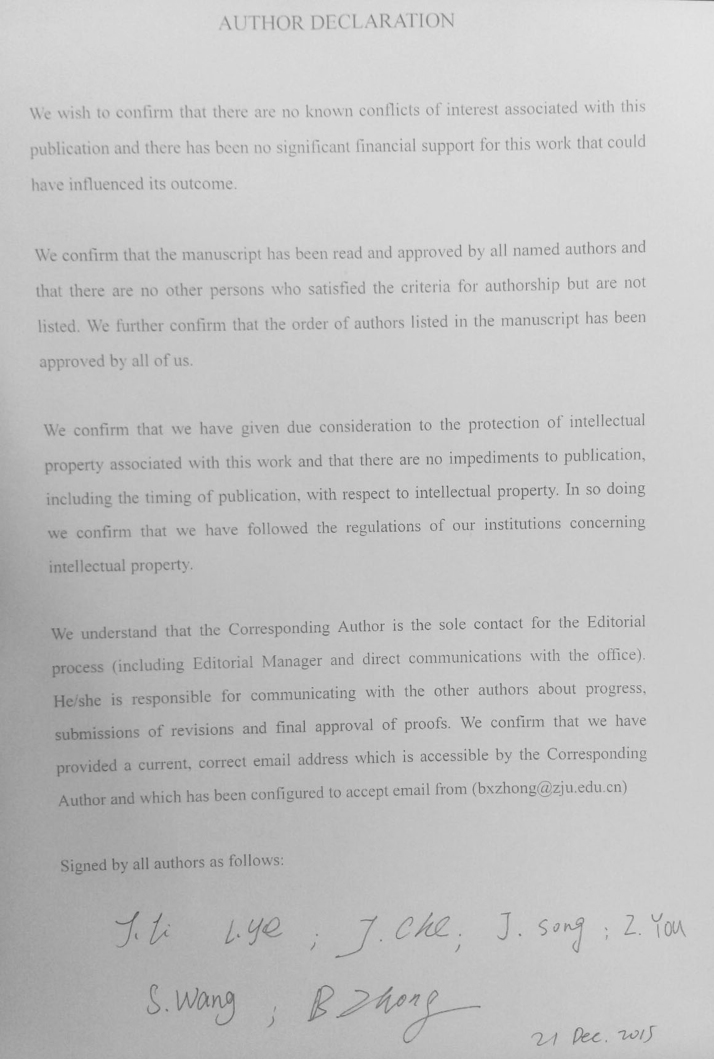


**Fig. 1:**
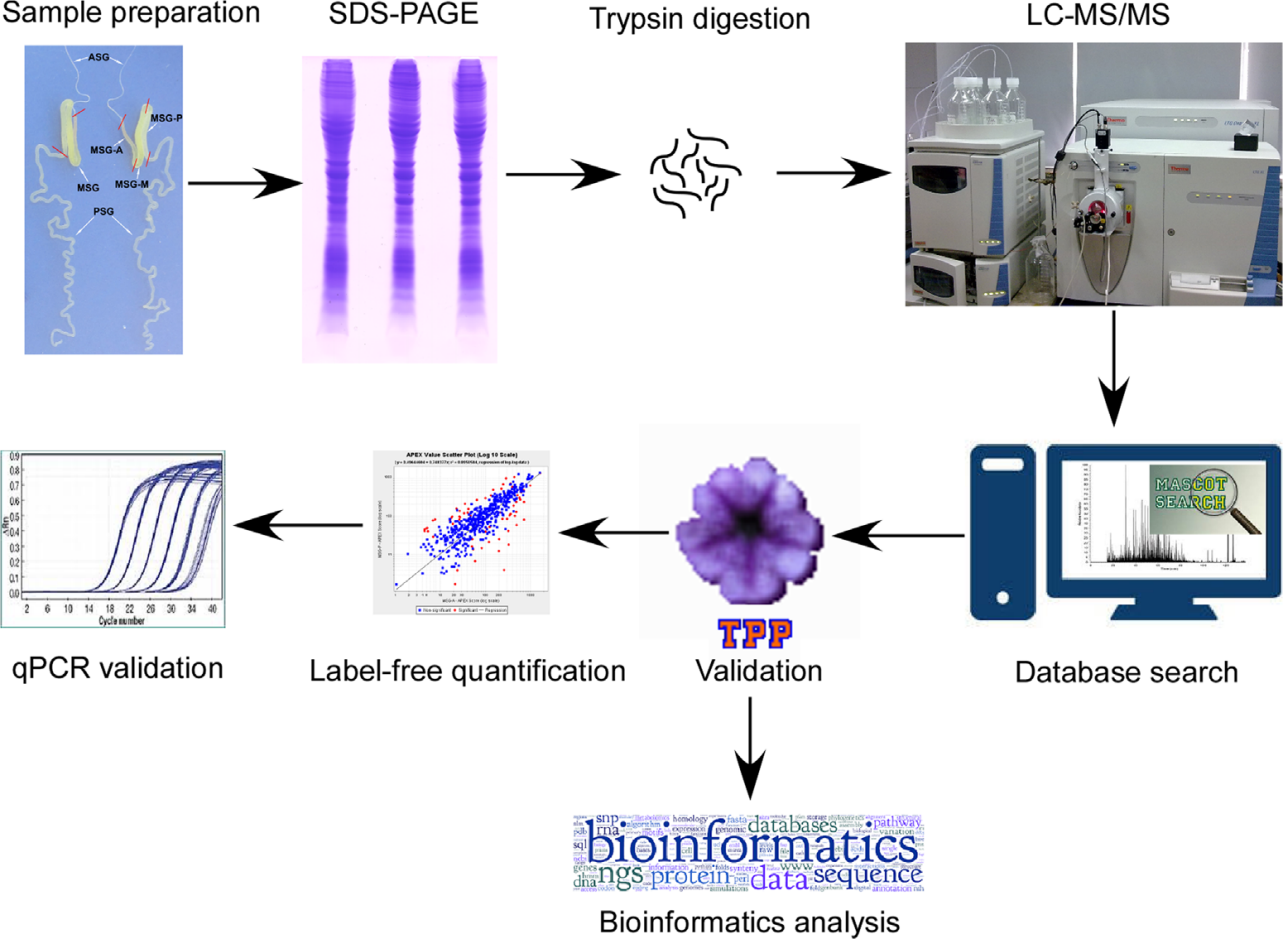
Workflow of the MSG proteome identification and data processing.
